# Manejo de muestras lipémicas en el Laboratorio Clínico

**DOI:** 10.1515/almed-2022-0083

**Published:** 2023-02-20

**Authors:** Carla Fernández-Prendes, María J. Castro Castro, Lourdes Sánchez Navarro, Loreto Rapún Mas, Cristian Morales-Indiano, Teresa Arrobas Velilla

**Affiliations:** Servicio de Anàlisis Clínics i Bioquímica, Laboratori Clínic Metropolitana Nord, Hospital Germans Trias i Pujol, Badalona, España; Comisión de lipoproteínas y enfermedades cardiovasculares, Sociedad Española de Medicina del Laboratorio, Barcelona, España; Servicio de Bioquímica Clínica, Laboratori Cliníc Territorial Metropolitana Sud, Hospital Universitari de Bellvitge, L’Hospitalet de Llobregat, España; Comisión de Biología Hematológica, Sociedad Española de Medicina de Laboratorio, Barcelona, España; Laboratorio de Nutrición y Riesgo Cardiovascular, Hospital Universitario Virgen Macarena, Sevilla, España

**Keywords:** índice lipemia, interferencia, íntralipid, índices séricos, lipemia

## Abstract

Las interferencias analíticas en el laboratorio clínico pueden causar errores en la interpretación de los resultados de diversas magnitudes biológicas por parte del médico peticionario. Las interferencias analíticas más frecuentemente observadas en el laboratorio clínico son la hemólisis, ictericia y lipemia. La lipemia se define como la turbidez de la muestra causada por la acumulación de lipoproteínas, principalmente lipoproteínas de muy baja densidad (VLDL) y quilomicrones. Existen diversos métodos de detección de muestras lipémicas, como por ejemplo, el índice lipémico o la determinación de triglicéridos en muestras de suero o plasma o la Concentración de Hemoglobina Corpuscular Media (CHCM) en muestras de sangre. Las empresas de diagnóstico *in vitro* son las responsables, según la Directiva Europea 98/79/CE, de realizar el estudio de las sustancias interferentes que pueden afectar a la medición de una magnitud. Existe una necesidad urgente de estandarizar la forma en que se realizan y se reportan los estudios de interferencia por parte de los fabricantes. La interferencia por lipemia puede ser eliminada por diferentes métodos permitiendo la determinación de magnitudes biológicas de manera exacta. El laboratorio clínico debe decidir los protocolos de actuación ante muestras lipémicas dependiendo de la magnitud biológica que se quiere analizar.

## Introducción

Las interferencias analíticas en el laboratorio clínico causan cambios analíticamente significativos en los resultados de las magnitudes biológicas, ocasionalmente estos cambios pueden llegar a ser clínicamente significativos, conduciendo a interpretaciones erróneas por parte del médico peticionario.

Una de las interferencias analíticas más frecuentemente observadas en el laboratorio es la lipemia [1]. La lipemia se define como la turbidez de la muestra causada por la acumulación de lipoproteínas, principalmente lipoproteínas de muy baja densidad (VLDL) y quilomicrones, es decir, partículas compuestas por un alto porcentaje en triglicéridos.

Las lipoproteínas presentan una alta heterogeneidad de tamaño y no todas contribuyen por igual a la turbidez. Los quilomicrones son las partículas lipoproteicas de mayor tamaño (70–1000 nm) y las que más turbidez causan en la muestra. Las lipoproteínas de muy baja densidad (VLDL) existen en tres clases de tamaño; pequeño (27–35 nm), intermedio (35–60 nm) y grande (60–200 nm). Sólo las VLDL intermedias y grandes producen turbidez. Las lipoproteínas de alta densidad (HDL) (6–12,5 nm) y lipoproteínas de baja densidad (LDL) (20–26 nm) no producen turbidez en la muestra [2, 3].

La frecuencia de muestras lipémicas oscila entre 0,5 y 2,5% dependiendo de las características del hospital y la proporción de pacientes hospitalizados y de Atención Primaria. Además, la hemólisis de la muestra aumenta cuando los eritrocitos se mantienen suspendidos en plasma lipémico, por lo que la frecuencia de hemólisis se incrementa con el aumento de la lipemia [4].

La causa preanalítica más común de lipemia es el no ayuno del paciente, sin embargo, se ha demostrado en recientes estudios que la falta de ayuno no produce cambios clínicamente significativos en la concentración de lipoproteínas, considerando la necesidad de ayuno, solamente cuando los triglicéridos sin ayuno sean mayores de 400 mg/dL (4,56 mmol/L) [5].

La lipemia grave capaz de causar una interferencia significativa puede ocurrir en hipertrigliceridemias primarias (síndrome de quilomicronemia familiar) o secundarias (diabetes mellitus, resistencia a la insulina, alcoholismo, infecciones por virus de inmunodeficiencia humana, enfermedad renal, etc.) [1]. Las nutriciones parenterales y los diluyentes para fármacos poco solubles en agua que contienen emulsiones lipídicas también pueden producir lipemia [6].

## Objetivo y campo de aplicación

El objetivo de este documento es proporcionar información sobre la interferencia provocada por la lipemia en la determinación de diferentes magnitudes biológicas y la creación de un protocolo de actuación común para todos los laboratorios clínicos ante la presencia de muestras de pacientes lipémicas.

El campo de aplicación es el manejo de muestras lipémicas en los laboratorios clínicos, incluyendo detección de la lipemia, reglas de ampliación de pruebas, diferentes métodos de extracción de lípidos e informe de resultados.

## Interferencia por lipemia

La interferencia analítica se define como el cambio significativo en la medición de una magnitud biológica debido a la presencia de una sustancia interferente en la muestra.

La interferencia se puede investigar comparando el método a estudio con un método no afectado por dicha interferencia. Actualmente no se dispone de métodos analíticos no interferidos por lipemia para la mayoría de las magnitudes bioquímicas, por lo que estos estudios se realizan utilizando diferentes procedimientos: agregando lípidos artificiales que intentan simular la turbidez de los lípidos endógenos [7] o realizando una extracción de lípidos en muestras lipémicas de paciente a través de ultracentrifugación [8].

Los fabricantes de reactivos son responsables, según la Directiva Europea 98/79/CE [9], de realizar estudios de interferencias, proporcionando un límite de error máximo admisible.

La mayoría de los proveedores de reactivos utilizan Intralipid^®^ para simular la lipemia en muestras de suero, una emulsión lipídica para perfusión intravenosa que contiene aceite de soja purificado, fosfolípidos de huevo purificados y glicerol. Además, consideran la interferencia significativa cuando el resultado de la medición de la magnitud biológica antes y después de añadirle una concentración determinada de lípidos artificiales se desvía un 10% [10].

Por lo tanto, los estudios de interferencia que realizan los fabricantes tienen varias limitaciones: (a) no se tiene en cuenta la variabilidad biológica de las magnitudes estudiadas, (b) la composición de los lípidos artificiales y los endógenos difieren, mostrando resultados discordantes entre muestras lipémicas de pacientes y muestras de suero suplementadas con Intralipid^®^ [10], [11], [12], (c) actualmente no existen materiales estandarizados que simulen adecuadamente la lipemia y (d) si la composición de los lípidos artificiales contiene glicerol, éste interfiere en la medición de triglicéridos, ya que los analizadores automatizados que se utilizan generalmente para la cuantificación de triglicéridos no realizan un blanco de glicerol [13].

Además, el límite de error máximo admisible reportado por los fabricantes varía en función del instrumento, método analítico y criterio del fabricante, Roche Diagnostics y Beckman Coulter, ambos estudian la interferencia por lipemia a través de la adición de Intralipid^®^ a muestras de suero pero existen diferencias a la hora de informarlo (Tabla 1).

**Tabla 1:** Interferencias por lipemia en diversas magnitudes biológicas declaradas por dos fabricantes, Roche Diagnostics y Beckman Coulter.

Magnitudes biológicas	Roche Diagnostics	Beckman Coulter
	Intralipid^®^, mg/dL	
Srm—Alanina-aminotransferasa; c.cat.	150	300
Srm—Albúmina; c.masa	550	800
Srm—Aspartato-aminotransferasa; c.cat.	150	300
Srm—alfa-Amilasa; c.cat.	1500	1000
Srm—Apolipoproteína A-I; c.masa	1000	1000
Srm—Bilirrubina; c.sust.	1000	1000
Srm—Bilirrubina (esterificada); c.sust.	750	1000
Srm—Calcio (II); c.sust.	1000	1000
Srm—Cloruro; c.sust.	2000	500
Srm—Colesterol; c.sust.	2000	1000
Srm—Colesterol-HDL; c.sust.	2000	900
Srm—Creatinina; c.sust.	2000	1000
Srm—Creatina-cinasa; c.cat.	1000	1000
Srm—Factores reumatoides; c.sust.arb. (OMS 64/2)	2000	750
Srm—Ferritina; c.masa	1000	1000
Srm—Fosfatasa alcalina; c.cat.	2000	1000
Srm—Fosfato; c.sust.	800	800
Srm—gamma-Glutamiltransferasa; c.cat.	2000	1000
Srm—Glucosa; c.sust.	1000	700
Srm—Haptoglobina; c.masa	600	1000
Srm—Hierro (II + III); c.sust.	1500	100
Srm—Inmunoglobulina A; c.masa	2000	1000
Srm—Inmunoglobulina G; c.masa	2000	1000
Srm—Inmunoglobulina M; c.masa	2000	200
Srm—Litio; c.sust.	2000	2000
Srm—Magnesio (II); c.sust.	2000	200
Srm—Potasio; c.sust.	2000	500
Srm—Proteína; c.masa	200	1000
Srm—Proteína C reactiva; c.masa	1000	1000
Srm—Sodio; c.sust.	150	500
Srm—Transferrina; c.sust.	500	1000
Srm—Urea; c.sust.	1000	500
Srm—Urato; c.sust.	1500	1000

La realización del estudio de interferencias utilizando la ultracentrifugación para eliminar los lípidos en muestras lipémicas de pacientes también tiene sus limitaciones. Debido a la gran heterogeneidad en el tamaño y cantidad de las lipoproteínas presentes en las muestras lipémicas de pacientes, la desviación producida en la medición de las magnitudes biológicas debido a la lipemia es incierta. Es necesario realizar los estudios con un tamaño de muestra lo suficientemente grande para que sea representativo de toda la población [14].

Es evidente que existe una necesidad urgente de estandarizar la forma en que se realizan y se reportan los estudios de interferencia por parte de los fabricantes y los laboratorios deben ser conocedores de la posible falta de replicabilidad [15].

Los fabricantes de reactivos no realizan los estudios de interferencia en líquidos biológicos, debido a la gran variedad de matrices existentes. Sin embargo, en líquidos serosos es posible la presencia de altas concentraciones de lípidos que produzcan turbidez en la muestra.

El análisis bioquímico de los líquidos biológicos es clínicamente relevante en determinadas situaciones, por ejemplo, los criterios de Light comparan la concentración de proteínas totales del líquido pleural y lactato deshidrogenasa con mediciones séricas para diferenciar entre derrames de tipo exudado y trasudado, lo que da como resultado diferentes orientaciones diagnósticas. El cálculo del gradiente de albúmina en derrames peritoneales se basa en la medición de albúmina en líquido ascítico y suero para determinar si la ascitis es el resultado de una hipertensión portal. Otras pruebas clínicamente relevantes en los fluidos corporales incluyen medir el colesterol y los triglicéridos en el líquido pleural para detectar quilotórax y pseudoquilotórax [16], glucosa para derrames infecciosos o malignos y amilasa para detectar fístulas pancreáticas postoperatorias.

## Mecanismo de la interferencia por lipemia

La interferencia causada por la lipemia se debe principalmente a tres mecanismos distintos: la dispersión de la luz, el efecto del desplazamiento de volumen y la falta de homogenización de la muestra [10].

### Dispersión de la luz por las lipoproteínas

El principal mecanismo por el cual la lipemia produce interferencia en la medición de diversas magnitudes es la dispersión de la luz producida por las lipoproteínas (principalmente quilomicrones y VLDL). La lipemia provoca la dispersión de la luz en todo el espectro visible (300–700 nm), aumentando a medida que disminuye la longitud de onda. Los ensayos colorimétricos con lecturas de absorbancia en las longitudes de onda más cortas del espectro visible son, por lo tanto, más susceptibles a la interferencia [10]. El signo y la magnitud de la interferencia en los métodos espectrofotométricos dependen de si el método mide un aumento o disminución de absorbancia y de la longitud de onda utilizada. Por este motivo, es posible que la interferencia por lipemia no sea comparable en dos métodos analíticos diferentes (Tabla 1). Este mecanismo afecta a métodos espectrofotométricos, nefelométricos y turbidimétricos.

El tamaño y la composición de las lipoproteínas influyen en la dispersión de la luz, tanto los quilomicrones como las VLDL poseen tamaños muy heterogéneos contribuyendo de manera diferente a la turbidez de la muestra [2, 3].

### Efecto de desplazamiento de volumen

Este mecanismo afecta fuertemente a la medición del sodio. También afecta a otros electrolitos como potasio y cloruro, aunque la interferencia raramente es clínicamente significativa.

La mayoría de los analizadores automatizados determinan la concentración de los electrolitos mediante potenciometría indirecta utilizando electrodos selectivos de iones (ISE), para ello, realizan una dilución de la muestra, normalmente, de 1:20 a 1:34. El resultado se obtiene con un cálculo que se basa en la composición acuosa de la matriz del suero o plasma.

El suero o plasma se compone aproximadamente de un 92% de fase acuosa y un 7% de fase sólida. Tanto en las muestras lipémicas como en muestras de pacientes con hiperproteinemia, la fase acuosa disminuye y la medición de la concentración de los componentes que se distribuyen en esta fase, como los electrolitos, se infraestima. A este efecto se le denomina “efecto de desplazamiento de volumen” [10, 17], [18], [19].

### Falta de homogenización de la muestra

Este efecto se produce por la diferencia en la densidad de las partículas de la muestra de suero o plasma. Tras la centrifugación de la muestra, los quilomicrones y VLDL se posicionarán en la parte superior del tubo por su baja densidad, el resto de los constituyentes se distribuirán según su polaridad; los componentes hidrófobos en la fase lipídica (parte superior del tubo) y los hidrófilos en la fase acuosa (parte inferior del tubo).

Los analizadores disponen de unos sensores para evitar la colisión de la aguja con el tubo al pipetear la muestra, por esta razón, la toma de muestra se realiza mayoritariamente de la parte superior del tubo. Esto puede resultar en una falsa disminución de la concentración de componentes hidrófilos, al contrario de las sustancias hidrófobas (ácido valproico u hormonas esteroides) que se acumulan en la capa superior de lípidos [2, 3, 10].

### Otros mecanismos de interferencia por lipemia

La acumulación de lipoproteínas en la muestra de suero puede interferir con las magnitudes biológicas medidas por interacciones físicas y químicas. Esto es especialmente importante en los métodos electroforéticos [20].

La lipemia también puede interferir en algunos inmunoensayos, las lipoproteínas pueden interferir con la reacción antígeno-anticuerpo bloqueando los sitios de unión de los anticuerpos [21].

Asimismo, la presencia de lipemia puede producir alteraciones morfológicas en la población de neutrófilos; aumento de tamaño, desgranulación y vacuolización citoplasmática [22]. La elevada concentración de triglicéridos podría modular la señalización celular de los neutrófilos, aumentando su activación, adhesión y permeabilidad de la membrana [23]. Estas alteraciones morfológicas podrían interferir en la identificación de neutrófilos en analizadores que utilizan la dispersión de luz para caracterizar el diferencial leucocitario. La presencia de lipemia produce un aumento del tamaño de los neutrófilos pudiéndose solapar con la población de monocitos en las gráficas de dispersión leucocitaria. La lipemia puede producir alteración en la definición de los bordes de los eritrocitos, mostrándose borrosos en el frotis de sangre. Este hallazgo morfológico puede proporcionar información útil cuando no se sospeche la presencia de una hipertrigliceridemia y no se disponga de otros resultados del laboratorio.

En función del tipo de lipoproteína presente en la muestra, el recuento de leucocitos totales y de plaquetas podría estar falsamente elevado. Esta interferencia puede observarse tanto en analizadores que determinan el recuento de leucocitos y plaquetas por impedancia o bien por métodos ópticos, siendo en estos últimos más pronunciada la interferencia. Esto se debe a que las lipoproteínas poseen un alto índice de refracción pudiendo generar señales anormales en los canales de plaquetas y/o leucocitos. Además, la concentración de hemoglobina medida por métodos espectrofotométricos en los analizadores hematológicos puede proporcionar resultados falsamente elevados en presencia de una alta concentración de triglicéridos [24].

## Metodología para la medición de índices de lipemia

La detección visual de la lipemia en las muestras de pacientes sigue siendo una forma ampliamente utilizada, sobre todo en laboratorios con un bajo número de muestras, aunque este procedimiento visual es subjetivo, arbitrario y no suficientemente sensible. La turbidez producida por la lipemia, generalmente, se observa en muestras de suero o plasma con concentraciones de triglicéridos superiores a 300 mg/dL (3,42 mmol/L) [25]. Además, la detección visual en las muestras de sangre total no es posible si no se separan las células por sedimentación o centrifugación.

Una alternativa a la detección visual sería la medición de la concentración de triglicéridos como evaluación del grado de lipemia o turbidez, pero esta estimación tiene sus inconvenientes. El grado de turbidez no se correlaciona con la concentración de triglicéridos ya que la proporción de triglicéridos puede ser diferente según el tipo de lipoproteína presente en la muestra [10, 26].

Actualmente, gracias al desarrollo de procedimientos de medida automatizados para la determinación de índices séricos, se ha facilitado mucho la implementación de estos índices de manera rutinaria en los laboratorios clínicos. Los índices séricos miden la presencia de los principales interferentes que pueden estar presentes en estas muestras: hemoglobina, bilirrubina y turbidez (principalmente debida a lipemia).

La medición de los índices séricos se basa en la dilución de la muestra con una solución salina o un tampón y la realización de cálculos de lecturas de absorbancia que proporcionan una representación semicuantitativa de los niveles de ictericia, hemólisis o lipemia presente en las muestras de suero o plasma. Algunos métodos utilizan una solución de cloruro de sodio (NaCl) al 0,9% como reactivo y realizan lecturas de absorbancia a diferentes longitudes de onda, permitiendo calcular el índice de lipemia. Las fórmulas de cálculo incluyen correcciones para compensar la superposición espectral [27].

Las lipoproteínas dispersan la luz alrededor de 700 nm, y por lo tanto esas longitudes de onda se utilizan para evaluar el grado de lipemia. Todavía existe una gran heterogeneidad entre fabricantes en longitudes de onda utilizadas, sin embargo casi todos usan combinaciones de dos o más longitudes de onda. Por ejemplo, en la serie AU (Olympus), Beckman Coulter usa 660/800, en la serie Cobas, Roche usa 660/700 y Architect, Abbott usa varias longitudes de onda (510/524; 572/604; 628/660 y 524/804) en un cálculo del grado de lipemia [28]. En el caso de los analizadores de Werfen, ACLTOP serie 50, la evaluación de la interferencia por lipemia se realiza mediante una medición de la absorbancia óptica de la muestra diluida a tres longitudes de onda distintas (405, 535 y 671).

El índice de lipemia se mide en unidades de lipemia, que son lineales hasta 2000 mg/dL (22,8 mmol/L) y está basado en el comportamiento óptico de Intralipid^®^, con el inconveniente de que Intralipid^®^ difiere en su composición con los lípidos naturales [10, 27].

Las ventajas de la detección automática del índice de lipemia son muchas: bajo coste, elevada velocidad de procesamiento de las muestras, mayor reproducibilidad y acortamiento del tiempo de respuesta. Sin embargo, también hay algunas desventajas, como por ejemplo, falsas elevaciones del índice de lipemia en muestras en las que está aumentada su turbidez por la presencia de otras moléculas como las paraproteínas [29, 30].

En la determinación de magnitudes hemostasiológicas existen analizadores que disponen de un modúlo preanalítico que nos informa sobre la presencia de lipemia. Los intervalos a partir de los cuales las magnitudes se ven interferidas por lipemia están definidos por los propios fabricantes sin margen de modificación. Los analizadores emitirán una alarma cuando el índice de lipemia supere el umbral definido en cada magnitud. Algunos analizadores que no disponen del módulo preanalítico emiten una alarma de sospecha por lipemia, en estos casos se debe comprobar visualmente.

En sangre total no existen unos índices que nos informen sobre la calidad de la muestra. La detección de lipemia en sangre total es difícil y se recurre a la Concentración de Hemoglobina Corpuscular Media (CHCM) como índice más sensible de su presencia. Se estima que un CHCM >360 g/L (>36 g/dL), descartando otras posibles causas como anemia hemolítica o aglutininas, puede ser debido a la presencia de lipemia que interfiere produciendo un aumento en la concentración de hemoglobina [24].

La determinación de hemoglobina se realiza por espectofotometría (normalmente medida a 425 nm) y su falso incremento se debe a la turbidez generada por la lipemia. Al estar afectada la concentración de hemoglobina, los índices eritrocitarios que dependen de su resultado también se verán afectados, aumentando falsamente la Hemoglobina Corpuscular Media (HCM) y la Concentración de Hemoglobina Corpuscular Media (CHCM).

Algunos analizadores de hematimetría disponen de método ópticos de dispersión láser en el canal de reticulocitos como alternativa para la cuantificación de hemoglobina. Los autoanalizadores de hematimetría que disponen de los dos métodos emiten una alarma de posible interferencia por lipemia cuando existen diferencias entre el valor de hemoglobina por fotometría y por dispersión óptica. Estos analizadores también utilizan la diferencia entre el CHCM (hemoglobina medida por fotometría) y el CHCM calculado (hemoglobina medida por dispersión óptica) para detectar la presencia de interferencia. Una de las limitaciones que presenta dichas magnitudes es que no son reportables. Algunas gráficas como los histogramas de leucocitos por impedancia, los gráficos de dispersión de las subpoblaciones leucocitarias (flourescencia, dispersión làser) o los que determinan la presencia de eritroblastos (dispersión làser) pueden mostrar alarmas que indiquen la posible presencia de lipemia en la muestra.

## Métodos de aclaramiento de muestras lipémicas

### Métodos de centrifugación

La centrifugación de las muestras de suero o plasma es el procedimiento de elección para el tratamiento de la lipemia. Las muestras se ultracentrifugan (100.000–2.000.000×*g*) eliminando eficazmente los lípidos y permitiendo la medición de las magnitudes analíticas [31, 32]. La ultracentrifugación requiere tener un equipo que no está disponible en la mayoría de laboratorios. Sin embargo, se puede obtener una separación de lipoproteínas de elevado tamaño, quilomicrones, en muestras de suero o plasma realizando centrifugaciones de alta velocidad (10.000–15.000×*g*) [33, 34]. Si la lipemia es causada por acumulación de VLDL, el proceso es menos efectivo.

Tras la centrifugación de alta velocidad, una capa de lípidos queda en la parte superior, se recupera el infranadante con una pipeta de vidrio con cuidado de no contaminar la muestra con la capa lipídica. Se realiza la medición de las magnitudes bioquímicas en el infranadante. Este método de aclaramiento no sería apropiado para la medición de sustancias hidrofóbicas (hormonas, fármacos, etc.) ya que se distribuirán en la capa de lípidos, y la medición en el infranadante provocará una disminución falsa del resultado.

### Métodos de extracción

La extracción de lípidos en muestras de suero o plasma se puede realizar con solventes polares, como por ejemplo el 1,1,2-triclorotrifluoroetano.

Lipoclear^®^ es un polímero no iónico que se ha utilizado mucho en el laboratorio clínico para el aclaramiento de muestras lipémicas, pero en estos momentos no se encuentra disponible en el mercado.

El hecho de adicionar una sustancia exógena a la muestra puede causar interferencias en la medición de las magnitudes bioquímicas, como es el caso de la medición de las concentraciones de proteínas, calcio y aspartato-aminotransferasa cuando se tratan las muestras con 1,1,2-triclorotrifluoroetano o proteínas, albúmina y calcio cuando se tratan las muestras con Lipoclear^®^ [35].

### Métodos de dilución o de reemplazo

La dilución de muestras de suero o plasma para la eliminación o la disminución de la concentración de un interferente podría ser un método adecuado, sobre todo para la medición de aquellas magnitudes analíticas que son lipófilas y su distribución en la fase lipídica es importante. La muestra debería ser diluida a un factor de dilución suficiente para disminuir el índice de lipemia por debajo del límite de interferencia de las magnitudes bioquímicas en cuestión, pero teniendo en cuenta no disminuir más abajo del límite de cuantificación de dichas magnitudes interferidas [2].

El reemplazo isovolumétrico del plasma con diluyente isoosmótico representa un método adecuado para la disminución de la interferencia en el caso de las muestras de hematimetría. Pese a ello, también hay que tener presente que pueden producirse resultados erróneos al arrastrar parte de las células en ese reemplazo.

### Métodos de medida alternativos

La potenciometría directa o la amperometría, métodos presentes en algunos analizadores de gases, se pueden utilizar para la medición de iones o creatinina, respectivamente. Las magnitudes hemostasiológicas como el tiempo de protrombina o el tiempo de tromboplastina parcial activada, se pueden determinar por métodos de detección del coágulo electromecánicos. Para la concentración de hemoglobina, algunos analizadores hematimétricos disponen de una segunda tecnología que permiten realizar el cálculo estimado de la concentración de hemoglobina a partir de un algoritmo derivado de la medición de los eritrocitos por citometría de flujo. El inconveniente de esta tecnología es que no está aprobada para su uso clínico.

## Protocolo para la detección y tratamiento de muestras lipémicas

Las estrategias de detección y manejo de muestras lipémicas son bastante heterogéneas en Europa. Actualmente, no existe un consenso de actuación ante la presencia de muestras lipémicas en el laboratorio clínico. El Grupo de Trabajo para la Fase Preanalítica de la Federación Europea de Química Clínica y Medicina de Laboratorio (EFLM) ha realizado una encuesta a 1416 laboratorios de 45 países europeos sobre el manejo de muestras lipémicas con el objetivo de utilizar los datos para proporcionar recomendaciones y armonizar los procesos.

Esta encuesta muestra que el 14% de los laboratorios no monitorizan la detección de lipemia. De los laboratorios que monitorizan la detección de lipemia, el 43% (n=493) realiza una medición automática de índices de lipemia, el 30% (n=348) inspección visual de muestras y el 28% (n=319) una combinación de ambos. Solamente el 25% (n=203) verifica la calidad de estas mediciones mediante controles de calidad internos. El 37% de los laboratorios realiza mediciones adicionales de triglicéridos en muestras lipémicas. La depleción de lípidos para eliminar la interferencia se realiza únicamente en el 27% de los laboratorios, siendo el método de aclaramiento más utilizado la centrifugación, seguido de la dilución y reactivos de extracción. El 72% de los laboratorios rechazan las magnitudes afectadas por lipemia, acompañadas de un comentario en el informe, el 21% publica los resultados de todas las magnitudes con un comentario de información general sobre la presencia de lipemia en la muestra y un 6,6% informa todos los resultados sin incluir ningún comentario en el informe [36].

**Figura 1:**
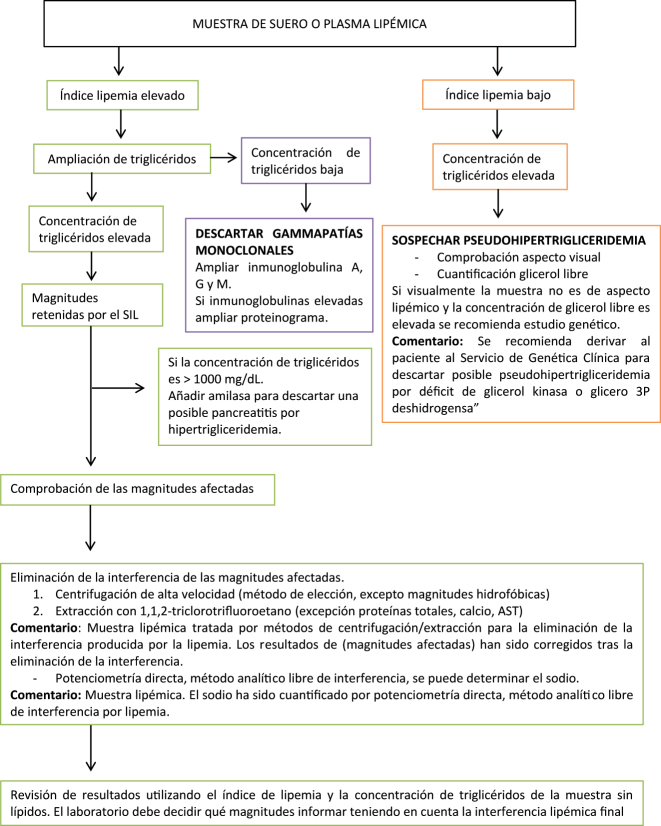
Diagrama de flujo para la detección y tratamiento de muestras de suero o plasma lipémicas en el análisis de magnitudes bioquímicas.

**Figura 2:**
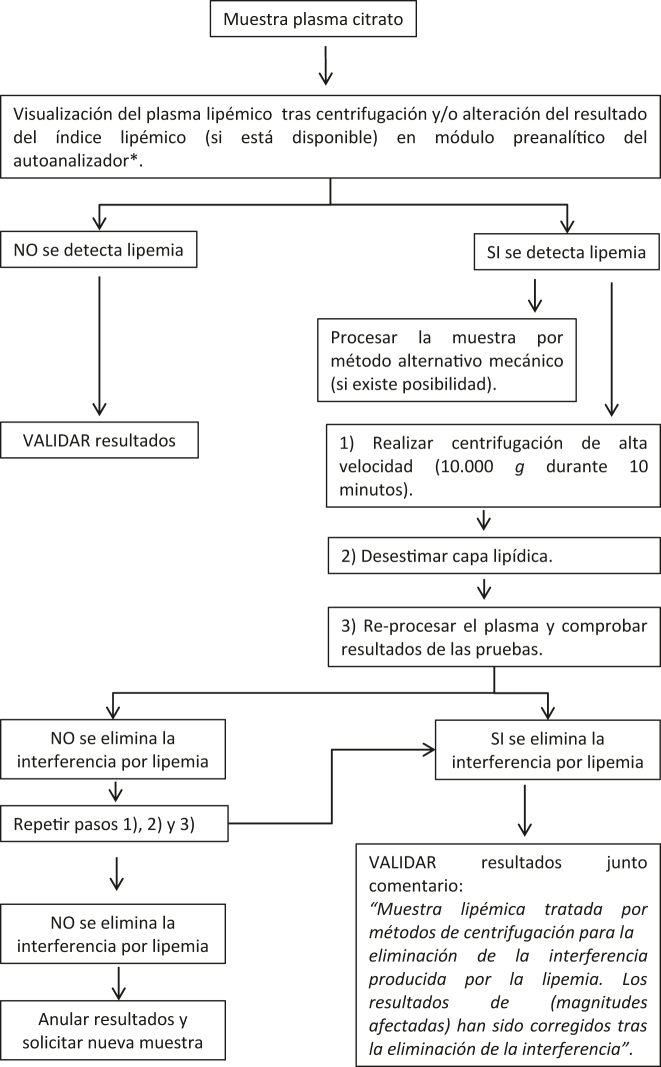
Diagrama de flujo para la detección y tratamiento de muestras de plasma citrato lipémicas en el análisis de magnitudes hemostasiológicas. *Método disponible en algunos analizadores de hemostasia.

**Figura 3:**
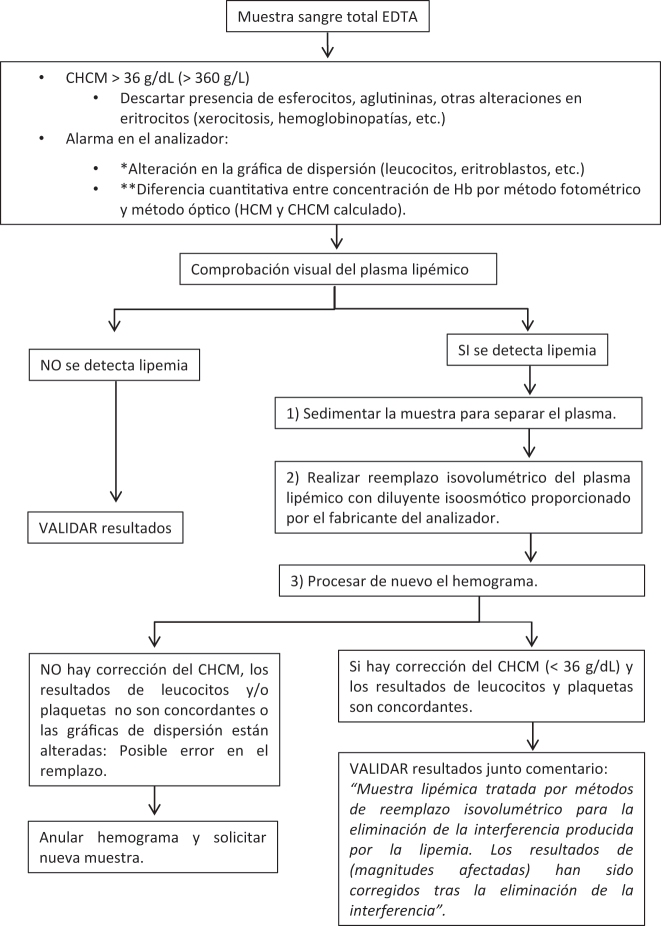
Diagrama de flujo para la detección y tratamiento de muestras de sangre total EDTA lipémicas en el análisis de magnitudes del hemograma. CHCM, Concentración de hemoglobina corpuscular media; HCM, Hhemoglobina corpuscular media; Hb, hemoglobina. *La gráfica alterada es específica de cada analizador hematológico. **Solo disponible en algunos analizadores hematológicos.

Con los resultados de esta encuesta europea, proponemos varios protocolos para la detección y el manejo de muestras lipémicas con el objetivo de lograr una armonización. Se proporcionan tres protocolos diferentes, con sus correspondientes diagramas de flujo, en función del tipo de muestra y magnitudes a analizar; magnitudes analíticas en suero o plasma (Figura 1), hemostasiológicas en plasma (Figura 2) y hematimétricas en sangre total (Figura 3).

### Magnitudes analíticas en suero o plasma

Las magnitudes interferidas por la lipemia deben ser retenidas en la validación en el sistema informático del laboratorio (SIL), mediante reglas que utilicen el índice de lipemia. Si el índice de lipemia es elevado añadir la determinación de triglicéridos mediante regla automatizada en el SIL.

Los dos métodos de detección de lipemia deben de utilizarse de manera complementaria, ya que cada uno tiene sus inconvenientes. El índice de lipemia está basado en el comportamiento óptico de Intralipid^®^ y la concentración de triglicéridos no se correlaciona con el grado de turbidez, ya que la turbidez varía según el tipo de lipoproteína presente en la muestra [26], [27], [28].

Cuando un resultado es retenido por el SIL se puede proceder de la siguiente manera:

– Comprobación de qué magnitudes pueden estar afectadas según el fabricante (Tabla 1) si el laboratorio no ha realizado un estudio de interferencia con muestras lipémicas de paciente (lípidos endógenos).
– Realizar una centrifugación a alta velocidad de la muestra (10.000* g*, 10 minutos) y reprocesamiento de la misma, método de elección para eliminar la interferencia por lipemia [33, 34]. Añadir un comentario en el informe de resultados “Muestra lipémica tratada por métodos de centrifugación para la eliminación de la interferencia producida por la lipemia. Los resultados de (magnitudes afectadas) han sido corregidos tras la eliminación de la interferencia”
– Analizar de nuevo el índice de lipemia y la concentración de triglicéridos. Si todavía existe interferencia, realizar el tratamiento de aclaramiento de muestras lipémicas con solventes como el 1,1,2-triclorotrifluoroetano [34, 35], reprocesar la muestra tratada y añadir comentario “Muestra lipémica tratada por métodos de extracción para la eliminación de la interferencia producida por la lipemia. Los resultados de (magnitudes afectadas) han sido corregidos tras la eliminación de la interferencia”
– Analizar de nuevo el índice de lipemia y la concentración de triglicéridos. El laboratorio decidirá qué magnitudes informar teniendo en cuenta la interferencia lipémica final.
– Determinar la concentración del sodio utilizando un analizador de gases con medición de iones mediante potenciometría directa [19] y añadir un comentario “Muestra lipémica. El sodio ha sido cuantificado por potenciometría directa, método analítico libre de interferencia por lipemia”.
– Se puede añadir la determinación de la concentración de amilasa en suero, en pacientes con triglicéridos >, 1000 mg/dL (11.4 mmol/L) ante la posible sospecha de pancreatitis por hipertrigliceridemia [37].
– En caso de triglicéridos elevados con índice de lipemia bajo hay que sospechar de pseudohipertrigliceridemia por aumento de glicerol basal, realizar una comprobación visual del aspecto de la muestra y la cuantificación del glicerol libre. Si el aspecto de la muestra no es lipémico y la concentración de glicerol libre es elevada se recomienda ampliar estudio genético, añadiendo un comentario en el informe de resultados “Se recomienda derivar al paciente al Servicio de Genética Clínica para descartar posible pseudohipertrigliceridemia por déficit de glicerol kinasa o glicero 3P deshidrogensa”. El glicerol presenta interferencia positiva con la determinación de triglicéridos debido a que la mayoría de los reactivos no realizan blanco de glicerol [38, 39].
– Si el índice de lipemia es elevado pero la concentración de triglicéridos es normal o baja, indica una alta posibilidad de presencia de paraproteínas en la muestra. Se recomienda añadir inmunoglobulina A, G y M. Si alguna de las inmunoglobulinas es elevada se puede añadir un proteinograma para descartar gammapatias monoclonales [40].

### Magnitudes hemostasiológicas en plasma

Ante la presencia de lipemia en una muestra para hemostasia se puede proceder de la siguiente manera [41], siguiendo este orden de prioridades:

– Visualización del plasma lipémico tras centrifugación o en caso de disponer de módulo preanalítico en el analizador comprobación del resultado del índice lipémico.
– Realizar la medición, en caso de disponer, por método alternativo mecánico.
– Si no se dispone de metodología mecánica realizar centrifugación de alta velocidad (10.000×*g*, 10 minutos). Desestimar la capa lipídica y reprocesar el plasma.
– Si no se consigue eliminar la interferencia por lipemia repetir el proceso de centrifugación de alta velocidad (10.000×*g*, 10 minutos).
– Una vez eliminada la interferencia, validar los resultados de las magnitudes hemostasiológicas añadiendo un comentario “Muestra lipémica tratada por métodos de centrifugación para la eliminación de la interferencia producida por la lipemia. Los resultados de (magnitudes afectadas) han sido corregidos tras la eliminación de la interferencia”

### Magnitudes hematimétricas

En hemogramas con valores de CHCM >36 g/dL, posiblemente debidos a la interferencia por lipemia, se recomienda proceder de la siguiente manera:

Descartar presencia de esferocitos (indicativos de anemia hemolítica) mediante observación del frotis de sangre periférica. *Nota: La determinación de reticulocitos y magnitudes bioquímicas de hemolisis (haptoglobina, bilirrubina, lactato deshidrogenasa) completan el diagnóstico de anemia hemolítica.*

Comprobación visual de la muestra tras sedimentación. Si el aspecto del plasma no es lipémico (y no hay sospecha de esferocitos por hemolisis) proceder calentado la muestra (37 °C, 30 minutos) y reprocesar. Si la interferencia no desaparece aumentar el tiempo a 1hora o incluso 90 min. Este efecto puede ser debido a las aglutininas presentes en la muestra.

Si el aspecto del plasma es lipémico proceder de la siguiente manera:

Realizar el reemplazo isovolumétrico del plasma con diluyente isoosmótico proporcionado por el fabricante de los analizadores utilizados y reanalizar el hemograma. Si la determinación del CHCM es inferior a 36 g/dL, verificar la correcta realización del remplazo isovolumétrico, comprobando que los resultados de leucocitos y de plaquetas sean concordantes con los obtenidos de la muestra original. El resto de magnitudes del hemograma y las gráficas proporcionadas por el analizador no deben presentar alteraciones. Validar los resultados con un comentario “Muestra lipémica tratada por métodos de reemplazo isovolumétrico para la eliminación de la interferencia producida por la lipemia. Los resultados de (magnitudes afectadas) han sido corregidos tras la eliminación de la interferencia”

Si tras el reemplazo isovolumétrico del plasma no se consiguen unos resultados apropiados del hemograma (posible error en el reemplazo), se debe anular el hemograma indicándolo en un comentario y solicitando una nueva muestra.

## Conclusiones

Este artículo revisa los diferentes mecanismos de interferencia por presencia de lipemia, y las diferentes estrategias para intentar evitar esta interferencia con el objetivo de ser utilizado como guía para profesionales de laboratorio clínico.

Es importante realizar algún método semicuantitativo para la medición del índice de lipemia en las muestras de suero/plasma y la cuantificación de triglicéridos y tener presente las posibles interferencias en el método analítico utilizado para la medición de triglicéridos. En caso de concentración elevada de triglicéridos e índices de lipemia bajos, sospechar de pseudohipertrigliceridemia por aumento de glicerol basal.

La determinación de los índices séricos tiene sus limitaciones, se debe sospechar de presencia de paraproteínas cuando los índices séricos sean elevados con concentraciones de triglicéridos bajas.

Se pueden realizar centrifugaciones de alta velocidad como método de elección para el aclaramiento de muestras de suero o plasma y medir magnitudes bioquímicas, no lipófilas, o hemostasiológicas. Como método de segunda elección para las muestras de suero o plasma, realizar la extracción de lípidos mediante solventes polares (1,1,2-triclorotrifluoroetano).

En las magnitudes lipófilas medidas en plasma o suero se recomienda realizar dilución de la muestra y en las magnitudes hematológicas en sangre total reemplazo isovolumétrico con diluyente isoosmótico.
